# The role of CAR and SII values in determining mortality in COVID-19 patients: a retrospective observational study

**DOI:** 10.1186/s12879-026-13730-8

**Published:** 2026-06-05

**Authors:** Sedat Özbay, Orhan Özsoy, Tansu Gençer, Mustafa Korkut, Cihan Bedel, Gülsüm Kavalcı, Cemil Kavalcı

**Affiliations:** 1 Sivas Numune Hospital Department of Emergency Medicine, Sivas, Turkey; 2 Department of Emergency Medicine, Health Science University Antalya Training and Research Hospital, Kazım Karabekir Street, Muratpaşa, Antalya, 07100 Turkey; 3 Department of Anesthesia, Health Science University Antalya Training and Research Hospital, Antalya, Turkey

**Keywords:** COVID-19 mortality, Laboratory parameters, Emergency department

## Abstract

**Aim:**

This study aimed to determine the relationship between CAR, SII and mortality in patients with coronavirus disease (COVID-19).

**Methods:**

This retrospective study included COVID-19 patients admitted to the emergency department (ED) of a secondary unit care hospital. The relationship between the laboratory parameters (Neutrophil-lymphocyte ratio (NLR) and Platelete-lymphocyte ratio (PLR), fibrinogen, D-dimer, procalcitonin, ferritin, and C-reactive protein, blood urea nitrogen (BUN), creatinine (Crea)), and COVID-19 mortality was examined by comparison with the survival group. Logistic regression analysis was performed to investigate the independent factors affecting mortality.

**Results:**

A total of 1519 COVID-19 patients were analyzed. Mortality was observed in 799 patients (52.6%), and survival was observed in799 patients (52.6%). Neutrophil-lymphocyte ratio (NLR) and Platelete-lymphocyte ratio (PLR) averages were found to be higher and more significant in the mortality group compared to the survival group (*p* < 0.001). The levels of fibrinogen, D-dimer, procalcitonin, ferritin, and C-reactive protein (CRP), which are acute-phase reactants, were found to be higher and statistically significant in the mortality group than in the survival group (*p* < 0.001), and blood urea nitrogen (BUN), creatinine (Crea), aspartate aminotransferase (AST), and C-reactive protein (CRP) were found to be independent factors affecting mortality in patients with COVID-19 (*p* < 0.05).

**Conclusions:**

SII and CAR cannot be used as independent predictors of mortality in COVID-19 patients.

## Introduction

Approximately 4 years ago, Coronavirus-related disease (COVID-19), which was named “Severe Acute Respiratory Syndrome Coronavirus-2” (SARS-CoV-2) by the World Health Organization (WHO), caused the deaths of millions of people worldwide [1]. Approximately 80% of infected people have mild-to-moderate symptoms, and considering the risk factors in the remaining population, approximately 20% of infected individuals may develop a serious condition requiring hospitalization, which is the most serious complication in patients requiring hospitalization owing to diffuse alveolar damage [2, 3].

A comprehensive examination of the relationship between the C-reactive protein to albumin ratio (CAR) and systemic immune-inflammation index (SII) in relation to the prognosis of patients with severe acute respiratory syndrome coronavirus 2 (SARS-CoV-2) has been conducted, revealing their significant roles as prognostic markers. The CAR, calculated by dividing the C-reactive protein level by the albumin level, has been demonstrated to be elevated in patients with severe acute respiratory syndrome (SARS-CoV-2) who require admission to the intensive care unit (ICU) and in those who succumb to the disease, thus substantiating its value in predicting severe outcomes and mortality [4]. Concurrently, the SII, calculated by dividing the neutrophil and platelet counts by the lymphocyte count, has emerged as a novel prognostic marker. A substantial increase has been observed in patients with severe cases of SARS-CoV-2 infection and those in critical condition, indicating its potential as a diagnostic tool for assessing disease severity [5, 6]. A meta-analysis of existing studies lends further credence to the association between elevated SII levels at the time of admission and an increased risk of in-hospital mortality, particularly among patients initially categorized as low-risk [3]. The prognostic value of the SII is underscored by its capacity to predict critical illness and mortality, thereby rendering it a valuable instrument for early risk stratification in patients with SARS-CoV-2 infection [6, 7]. While both the Charlson Comorbidity Index (CCI) and the SII have been demonstrated to be effective in predicting the outcomes of patients infected with the novel coronavirus, studies have indicated that the SII may outperform other markers, such as the neutrophil-lymphocyte ratio (NLR), in certain contexts. However, the findings of these studies require further validation through larger-scale investigations. It has been demonstrated that both CAR and SII are integral components in understanding the inflammatory response in cases of severe acute respiratory syndrome coronavirus 2 (SARS-CoV-2) and can guide clinical decision-making by identifying patients at a higher risk of severe disease and mortality.

In this study, we aimed to determine the relationship between CAR, SII, and mortality in patients with coronavirus disease (COVID-19).

## Methods

### Study design and settings

This single-center, retrospective, observational study was conducted after receiving approval from the ethics committee of the Sivas Numune Hospital Emergency Department (ED) (251-59-10106-25-111/01). Patients diagnosed with COVID-19 in the ED between January 1, 2020, and December 31, 2021, were included in the study. Since the study was retrospective, informed consent was not obtained from the participants. The study was conducted in accordance with the Helsinki Declaration.

### Study population

Patients aged > 18 years who were diagnosed with COVID-19 after anamnesis, examination, and tests performed in the ED were included in this study. The study data were obtained by scanning patient information and results using a hospital information technology system. The system revealed that 1825 patients were diagnosed with COVID-19. Patients with incomplete data (*n* = 206) were excluded from the analysis. A total of 1519 patients were included in the study.

### Study protocol and interventions

Demographic information of the patients, such as age and sex, was recorded. Additionally, highly sensitive troponin, procalcitonin, D-dimer, fibrinogen, albumin, CRP, ferritin, AST, ALT, BUN, creatinine, Na, hemoglobin levels, leukocytes (WBC), neutrophils, lymphocytes, platelets (PLT), PLR, NLR, CAR, and SII of the patients at the time of admission to the emergency department were recorded. Patients were divided into two groups: those who died one month after being diagnosed (30-day mortality) with COVID-19 (group 1) and those who survived after diagnosis (group 2). SII= Platelet count×Neutrophil count/ Lymphocyte count. CAR = C-reactive protein (CRP)/ Albumin. Demographic and laboratory data of the groups were compared statistically.

### Statical analysis

Data were evaluated using the statistical package program IBM SPSS Statistics (version 23.0; IBM Corp., Armonk, New York, USA). Descriptive statistics are expressed as the number of patients (n), percentage (%), mean standard deviation (±), median, and interquartile range (IQR). The normal distribution of the quantitative variable data was evaluated using the Kolmogorov-Smirnov normality test. The Levene Test was used to evaluate the homogeneity of group variances. The Mann-Whitney U test was used to compare variables between the groups. Multivariate logistic regression analysis was performed to identify independent factors affecting mortality. Variables with a p-value of < 0.10 in the group comparison were transferred to the multiple model. In the study conducted using the backward stepwise method, statistical significance was set at *p* < 0.05and 95% CI.

## Results

A total of 1519 patients were included in this study. In terms of sex, 60.2% (*n* = 915) of the patients were male. The average age of all patients was 69.98 ± 15.06 years. Patients were categorized into two groups: mortality and survival. Mortality was observed in 799 patients (52.6%), and survival was observed in 720 patients (47.4%). Patient demographic data are shown in Table 1. The prognostic relationship of COVID-19-related laboratory parameters for disease progression was compared between the mortality and survival groups. Accordingly, when the hemogram parameters of the patients were examined, the average WBC and neutrophil counts were found to be higher in the mortality group than in the survival group [14.64 (12.16) vs. 9.69 (5.81), *p* < 0.001, 13.27(11.1) vs. 7.92(5.7), *p* < 0.001, respectively]. The lymphocyte count was lower in the mortality group than in the survival group [0.51(0.5) vs. 0.81 (0.6), *p* < 0.001]. The NLR and PLR averages were found to be higher in the mortality group than in the survival group [24.78(33.6) vs. 9.48(11.9), *p* < 0.001; 324.8 (377.3) vs. 280 (270.2), *p* < 0.001, respectively]. The levels of fibrinogen, D-dimer, procalcitonin, ferritin, and CRP, which is an acute-phase reactant, was higher in the mortality group than in the survival group (*p* < 0.001) (Table 2). The median albumin level was significantly lower in the mortality group than in the survival group (*p* < 0.001) (Table 2). In addition, the mean blood urea nitrogen, creatinine, Tropoin I and AST levels were significantly higher in the mortality group than in the survival group (*p* < 0.001) (Table 2). The median CAR and SII levels were significantly higher in the mortality group than in the survival group (*p* < 0.001) (Table 2). When the parameters affecting mortality were examined using logistic regression analysis, AST, BUN, and Crea were found to be associated with mortality in COVID-19 patients (Table 3).

**Table 1:** Baseline characteristics of patients

Variables	COVID-19 (*n* = 1519)
Age (years) mean ± SD	69.98 ± 15.06
Male gender n(%)	915 (60.2)
Female gender n(%)	604(39.8)
Mortality, n (%)	799 (52.6)
Survival n(%)	720(47.4)

**Table 2:** According to groups Baseline laboratory parameters of COVID-19 patients

	Mortality group (*n* = 799)	Survival group (*n* = 720)	*P* value
Age (years) (IQR)	74 (15)	71 (18)	< 0.001
**Laboratory test** (IQR)			
WBC count (×10^3^/mm^3^)	14.64 (12.16)	9.69 (5.81)	< 0.001
Hemoglobin (mg/dL)	10.7(3.1)	10.8(3.1)	0.900
PLT (×10^3^/mm^3^)	178 (160)	226 (157)	< 0.001
Neutrophil, (×10^3^/mm3)	13.27(11.1)	7.92(5.7)	< 0.001
Lymphocyte, (×10^3^/mm3)	0.51(0.5)	0.81 (0.6)	< 0.001
NLR	24.78(33.6)	9.48(11.9)	< 0.001
PLR	324.8 (377.3)	280 (270.2)	< 0.001
Fibrinogen (g/dl)	552(265)	470 (234)	< 0.001
D-dimer (ng/ml)	2742 (4252)	1342(2421)	< 0.001
Albumine, (mg/dL)	29.90 (5.4)	30 (5.4)	< 0.001
Procalcitonin, (mg/dL)	0.99(5.7)	0.15(0.4)	< 0.001
Ferritine, (mg/dL)	603.25 (249.1)	489.60 (368.9)	< 0.001
CRP (mg/dL)	127.80 (147.3)	35.60(99.6)	< 0.001
BUN(mg/dL)	131.4 (119)	52.4 (38)	< 0.001
Creatinine (mg/dl)	1.73(2.4)	0.68 (0.4)	< 0.001
Sodium (mg/dl)	144 (12)	139 (7)	< 0.001
Troponin I (IU/L)	108.85 (373.3)	14.40 (63.7)	< 0.001
ALT(IU/L)	51 (112)	36 (38)	< 0.001
AST(IU/L)	53.5 (165)	27 (18)	< 0.001
Lactate (mmol/L)	2.61 (1.67)	2.28 (1.7)	< 0.001
CAR	4.41 (5.4)	1.14 (3.3)	< 0.001
SII	3960.70(5708.4)	2192.42(3035.8)	< 0.001

WBC: white blood cell; CRP: C-reactive protein; BUN: Blood urea nitrogen PLT: Platelet count NLR: neutrophil-to-lymphocyte ratio; PLR: platelet-to-lymphocyte ratio; ALT: Alanine transaminase AST: Aspartate aminotransferase, SII; Systemic inflamatuar index, CAR; CRP/Albumine ratio

**Table 3:** Multivarite logistic regresion analysis of COVID-19 patients

	Odds ratio	*p*	CI 95%
Age (years) (IQR)	1.004	0.736	0.981-1.028
**Laboratory test** (IQR)			
WBC count (×10^3^/mm^3^)	1.027	0.855	0.772-1.367
Hemoglobin (mg/dL)	1.086	0.219	0.952-1.238
PLT (×10^3^/mm^3^)	0.999	0.427	0.995-1.002
Neutrophil, (×10^3^/mm3)	1.085	0.614	0.790 − 1.490
Lymphocyte, (×10^3^/mm3)	1.169	0.459	0.773-1.767
NLR	1.009	0.448	0.986-1.032
PLR	1.002	0.052	1.000-1.004
SII	1.000	0.111	1.000-1.004
CAR	1.251	0.469	0.682-2.297
Fibrinogen (g/dl)	1.000	0.829	0.998-1.002
D-dimer (ng/ml)	1.000	0.410	1.000–1.000
Albumine, (mg/dL)	0.975	0.628	0.878-1.082
Procalcitonin, (mg/dL)	1.058	0.093	0.991-1.131
Ferritine, (mg/dL)	0.999	0.372	0.998-1.001
CRP (mg/dL)	0.997	0.758	0.976–1.018
BUN (mg/dL)	1.016	**0.000**	1.008–1.024
Creatinine (mg/dl)	0.713	**0.029**	0.526-0.966
Sodium (mg/dl)	1.022	0.288	0.982-1.064
Hs-Troponin I (IU/L)	1.000	0.438	1.000–1.000
ALT (IU/L)	0.278	0.223	0.998-1.001
AST (IU/L)	1.004	**0.033**	1.000-1.008
Lactate (mmol/L)	1.091	0.407	0.888-1.339

WBC: white blood cell, CRP: C-reactive protein, BUN: Blood urea nitrogen, PLT: Platelet count, NLR: neutrophil-to-lymphocyte ratio, PLR: platelet-to-lymphocyte ratio, ALT: Alanine transaminase, AST: Aspartate aminotransferase, SII; Systemic inflamatuar index, CAR; CRP/Albumine ratio

## Discussion

This study aimed to determine the relationship between CAR and SII and mortality in patients with coronavirus disease (COVID-19). CAR and SII were not independent risk factors for mortality.

A variety of analyses of inflammation were conducted in the present study. Several studies have identified certain inflammatory markers that have been demonstrated to be valuable in predicting mortality and morbidity rates. Although it is known that many inflammatory parameters are indicators of systemic inflammation, NLR, one of the leading parameters, has been associated with mortality, especially in patients with sepsis. There are also publications showing that it is associated with mortality, especially in malignancy and cardiovascular and gastrointestinal diseases [8–10]. In our study, the neutrophil count was significantly higher in the mortality group. In contrast, the lymphocyte count was significantly lower in the mortality group. This relationship arises from the relationship between increased neutrophil and decreased lymphocyte counts, particularly in inflammatory diseases. Inflammation increases neutrophil stimulation in several viral diseases. However, the acceleration of lymphocyte apoptosis also suppresses lymphocytes [11]. Therefore, considering the results of our study, NLR has been shown to be an inflammatory parameter with a good capacity to predict mortality. In a similar study, City et al. showed that NLR was significant at a cut-off value of 9,1 in a study in which they studied the predictive role of NLR in COVID-19 mortality [12].

Our study also revealed the relationship between CRP level and mortality in COVID-19 patients as an important inflammatory marker. In summary, CRP, one of the most important inflammatory markers, is associated with the severity of inflammation and mortality. CRP levels have also been associated with disease severity, independent of age, sex, and physical condition. Studies on patients with COVID-19 have also reported that high CRP levels in the early period are an indicator of mortality [13, 14].

In patients diagnosed with coronary heart disease (CHD), a nonlinear relationship has been identified between cardiac all-cause mortality and cardiovascular-specific mortality. Higher CAR values have been associated with an increased risk of mortality, particularly before certain inflection points [15]. Conversely, CAR has been shown to be independently associated with long-term mortality in patients undergoing carotid artery stenting (CAS), thereby underscoring its potential value in risk stratification and treatment optimization [16]. In hypertensive patients, elevated CAR levels have been associated with a 60% increase in all-cause mortality risk, further substantiating its role in mortality prediction and management strategies [17]. In the context of aortic valve replacement (AVR), CAR has been demonstrated to predict short- and long-term mortality, with elevated CAR values signifying heightened risk, thereby functioning as a reliable prognostic instrument [18]. Critically ill patients with acute kidney injury (AKI) exhibit increased mortality associated with higher CAR levels, highlighting its importance in intensive care settings [19]. In patients diagnosed with chronic heart failure (CHF), CAR has been identified as an independent predictor of all-cause mortality, exhibiting superior prognostic value in comparison to CRP alone [20]. In addition, the present study has demonstrated that, in general, in ICU patients, CAR has exhibited considerable prognostic value for mortality, which is comparable to that of well-established scoring systems such as APACHE II [21]. In our study, the CAR rate was statistically significantly higher in the mortality group, but logistic regression analysis alone was found to be insufficient to predict mortality. In our study, we observed that patients with impaired kidney and liver function had a higher mortality rate. CAR ratio may be related to the severity of inflammation, but we believe that severe inflammation alone is not sufficient cause of mortality. Despite the well-documented prognostic value of CAR and SII in various clinical settings such as carotid artery stenting, appendicitis, and cardiovascular diseases, these markers did not emerge as independent predictors in our cohort. This discrepancy may be explained by the specific pathophysiology of severe COVID-19. While CAR and SII primarily reflect systemic inflammation, mortality in our study population appeared to be more strongly driven by markers of end-organ dysfunction, particularly renal and hepatic impairment. In multivariate analysis, blood urea nitrogen (BUN), creatinine, and aspartate aminotransferase (AST) remained independently associated with mortality, suggesting that once organ failure develops, it may outweigh the prognostic contribution of inflammatory indices. In contrast to conditions such as carotid artery disease or acute appendicitis—where inflammation plays a more central and isolated role in disease progression—COVID-19 is a complex, multisystem disease characterized by a transition from early inflammatory response to widespread organ damage. Therefore, although CAR and SII were significantly elevated in non-survivors, their effects may have been mediated through or overshadowed by downstream organ dysfunction. This interaction likely reduced their statistical independence in regression models.

A review of the extant literature reveals that SII has been identified as a significant biomarker. In a study conducted by Yıldız et al., the SII was identified as an important biomarker for complications occurring in patients with acute appendicitis [22]. In a study conducted by Bedel et al., SSI, an indirect measure of inflammation in patients with heart failure, was 50% sensitive and 94% selective in terms of patient mortality [23]. In a study by Yüksel et al., it was observed that the SII was prognostic in hypertensive conditions and inflammation in epistaxis patients presenting to the emergency department and that the SII value was significantly lower in non-hypertensive bleeding [24]. A study on patients with acute cholecystitis revealed a substantial increase in SII values in comparison with the control group, underscoring the potential of SII values as a complementary diagnostic marker to CRP in patient evaluation [25]. A review of the extant literature reveals that alterations induced by inflammation can be characterized using the SII, a methodology that offers indirect and cost-effective measurement. In our study, the SII was statistically significantly higher in the mortality group, but logistic regression analysis alone was found to be insufficient to predict mortality. In our study, we observed that patients with impaired kidney and liver function had a higher mortality rate. We believe that severe inflammation alone is not sufficient cause of mortality.

### Limitations

Our study had some limitations. First, it was a retrospective, single-center study. Our study had some limitations. Second, repeated serial measurements of inflammatory and blood parameters could not be performed. Another limitation of this study is the lack of data on patient comorbidities (e.g., hypertension, diabetes), smoking status, and obesity. One additional limitation is the omission of the interval between emergency department presentation and death from the logistic regression analysis.

## Conclusion

This study provides evidence that many different inflammatory parameters can be used as indicators of mortality in COVID-19 patients. According to our study results, SII and CAR cannot be used as independent predictors of mortality. It has been underlined that especially BUN, Crea and AST are independent indicators of mortality in COVID-19 patients.

## Data Availability

Availability of data and materials The datasets generated and/or analysed during the current study are available in the 438 supplementary material accompanying this manuscript.

## Data access statements

Research data supporting this publication are available from the corresponding authors.

## Abbreviations

COVID-19: Coronavirus-related disease
ED: Emergency department
NLR: Neutrophil/lymphocyte ratio
PLR: Platelet/lymphocyte ratio
AST: Aspartate aminotransferase
ALT: Alanine aminotransferase
BUN: Blood urea nitrogen
Crea: Creatinine
CRP: C-reactive protein
DBIL: Direct bilirubin
CAR: C-reactive protein/Albumine ratio
SII: Systemic immune inflammation index
